# Pre-transit vitamin C injection improves post-transit performance of beef steers

**DOI:** 10.1017/S1751731120000968

**Published:** 2020-10

**Authors:** E. L. Deters, S. L. Hansen

**Affiliations:** Department of Animal Science, Iowa State University, Ames, IA 50011, USA

**Keywords:** antioxidants, cattle, feedlot, oxidative stress, transportation

## Abstract

Although cattle can synthesize vitamin C (**VC**) endogenously, stress may increase VC requirements above the biosynthetic threshold and warrant supplementation. This study investigated the effects of a VC injection delivered before or after a long-distance transit event on blood parameters and feedlot performance of beef steers. Fifty-two days prior to trial initiation, 90 newly weaned, Angus-based steers from a single source were transported to Ames, IA, USA. On day 0, 72 steers (356 ± 17 kg) were blocked by BW and randomly assigned to intramuscular injection treatments (24 steers/treatment): saline injection pre- and post-transit (**CON**), VC (Vet One, Boise, ID, USA; 5 g sodium ascorbate/steer) injection pre-transit and saline injection post-transit (**PRE**) or saline injection pre-transit and VC injection post-transit (**POST**). Following pre-transit treatment injections, steers were transported on a commercial livestock trailer for approximately 18 h (1675 km). Post-transit (day 1), steers were sorted into pens with one GrowSafe bunk/pen (4 pens/treatment; 6 steers/pen). Steers were weighed on day 0, 1, 7, 30, 31, 56 and 57. Blood was collected from 3 steers/pen on day 0, 1, 2 and 7; liver biopsies were performed on the same 3 steers/pen on day 2. Data were analyzed as a randomized complete block design (experimental unit = steer; fixed effects = treatment and block) and blood parameters were analyzed as repeated measures. A pre-transit VC injection improved steer average daily gain from day 7 to 31 (*P* = 0.05) and overall (day 1 to 57; *P* = 0.02), resulting in greater BW for PRE-steers on day 30/31 (*P* = 0.03) and a tendency for greater final BW (day 56/57; *P* = 0.07). Steers that received VC pre- or post-transit had greater DM intake from day 31 to 57 (*P* = 0.01) and overall (*P* = 0.02) *v*. CON-steers. Plasma ascorbate concentrations were greatest for PRE-steers on day 1 and POST-steers on day 2 (treatment × day; *P* < 0.01). No interaction or treatment effects were observed for other blood parameters (*P* ≥ 0.21). Plasma ferric-reducing antioxidant potential and malondialdehyde concentrations decreased post-transit (day; *P* < 0.01), while serum non-esterified fatty acids and haptoglobin concentrations increased post-transit (day; *P* < 0.01). In general, blood parameters returned to pre-transit values by day 7. Pre-transit administration of injectable VC to beef steers mitigated the decline in plasma ascorbate concentrations and resulted in superior feedlot performance compared to post-transit administration.

## Implications

In the U.S. beef industry, cattle often change ownership and are transported between industry segments such as a cow-calf or stocker operation to a feedlot. Therefore, this research sought to determine whether pre- *v*. post-transit supplementation of a powerful antioxidant (vitamin C) would differentially affect feedlot performance. Based on improved performance of steers that received a pre-transit vitamin C injection, cow-calf or stocker operations may be able to generate premiums and increase demand for calves given injectable vitamin C prior to transport to a feedlot. Additionally, injectable vitamin C offers a good return on investment for cow-calf producers looking to retain ownership.

## Introduction

Due to the segmented nature of the U.S. beef industry, cattle will often be transported multiple times between birth and harvest. Transportation involves numerous stressors including feed and water deprivation, physical exertion and psychological stress that predispose cattle to poor health and performance upon arrival at the feedlot (Van Engen and Coetzee, 2018). Transit has been shown to decrease antioxidant status as well as increase biomarkers of oxidative stress and inflammation in cattle (Marques *et al.*, 2012; Deters and Hansen, 2020). Oxidative damage and inflammation are energetically expensive processes that have been associated with increased feedlot morbidity and decreased feedlot performance (Chirase *et al.*, 2004; Cooke, 2017). Ascorbate (VC) is the major water-soluble antioxidant present in plasma and tissues (Combs, 2008). Cattle are capable of synthesizing VC from glucose in the liver, and thus VC is not considered an essential dietary nutrient. Nevertheless, tissue and circulating concentrations of this nutrient have been shown to decrease in response to stress (Nakano and Suzuki, 1984; Padilla *et al.*, 2006). This suggests cattle may be unable to produce enough VC to meet their requirement during periods of stress, such as long-distance transit events. Injectable VC is a relatively easy and cost-effective method to rapidly increase VC status. However, excess VC is rapidly excreted when kidney reabsorption and tissue uptake mechanisms reach saturation (Toutain *et al.*, 1997), suggesting the timing of VC administration is vital to its effectiveness. This research sought to determine the effects of injectable VC before or after an 18- h transit event on blood parameters and feedlot performance of beef steers. It was hypothesized that an injection of VC prior to transit would mitigate the decline in antioxidant status and dampen the inflammatory response, subsequently improving feedlot performance over steers receiving no VC, while an injection of VC post-transit would simply replete antioxidant stores that were lost due to transit.

## Material and methods

### Animals and experimental design

Ninety unweaned, Angus-based steer calves (265 ± 14 kg) from a single source were purchased and transported approximately 645 km over a period of 7 h to the Iowa State Beef Nutrition Farm in October 2018. Three days after the arrival (day −49), steers received visual and electronic identification tags. Additionally, steers were vaccinated against clostridial (Vision 7; Merck Animal Health, Madison, NJ, USA) and respiratory (Vista Once SQ; Merck Animal Health) diseases and injected with doramectin (Dectomax; Zoetis, Parsippany, NJ, USA) for control of internal and external parasites. Steers were fed a common corn silage-based diet and housed in open dirt lots until day −17 when 80 steers were moved to partially covered concrete pens equipped with GrowSafe bunks (GrowSafe Systems Ltd, Airdrie, Alberta, Canada) for a pre-trial acclimation period. On day 0, the 72 steers most uniform in weight (356 ± 17 kg), disposition and health status were blocked by BW and randomly assigned to 1 of 3 treatments (24 steers/treatment): saline injection pre- and post-transit (**CON**), VC (Vet One; 250 mg sodium ascorbate/ml; 5 g/steer; 14.1 ± 0.7 mg/kg BW) injection pre-transit and saline injection post-transit (**PRE**) or saline injection pre-transit and VC injection post-transit (**POST**). The VC dose in the current study was chosen based on doses (mg/kg BW) reported by others who investigated the effects of injectable VC on cattle health (Cusack *et al.*, 2008; Urban-Chmiel *et al.*, 2011; Wilson *et al.*, 2015). Saline and VC treatments were delivered intramuscularly at a dose rate of 20 ml/steer (10 ml/injection site on opposite sides of the neck). Approximately 2 h after administration of pre-transit treatment injections (~1430 h) steers were loaded onto a commercial livestock trailer (Silverstar PSDCL-402; Wilson Trailer Company, Sioux City, IA, USA) and transported for approximately 18 h (1675 km), during which time steers did not have access to feed or water. The average loading density was 1.35 m^2^/steer which is in accordance with the minimum area allowances outlined in the Guide for the Care and Use of Agricultural Animals in Research and Teaching (Federation of Animal Science Societies, 2010). Steers were stratified by treatment to truck compartments to account for compartment variability.

On day 1, steers returned to the Iowa State University Beef Nutrition Farm (~0830 h), immediately received post-transit treatment injections and were sorted into pens equipped with one GrowSafe bunk/pen (4 pens/treatment; 6 steers/pen). From day 1 to 57, all steers were fed a common total mixed ration (**TMR**; Table 1) that was delivered once daily at approximately 0800 h and allowed *ad libitum* access to feed and water. Steers were weighed on day 0 (pre-transit) and 1 (post-transit) as well as prior to feeding on day 7, 30, 31, 56 and 57. To be consistent with the weighing conditions on day 1 and based on the average BW shrink due to transit, a 6% deduction was applied to all BW collected after day 1. Consecutive BW collected on day 30 and 31 and day 56 and 57 were averaged to determine mid- and final BW, respectively; these averages were used to calculate the average daily gain (**ADG**). Steer DM intake (**DMI**), ADG and feed efficiency (gain–feed, **G:F**) were calculated from day 1 to 7, 7 to 31, 31 to 57 and 1 to 57. On day 7, steers were implanted with 80 mg trenbolone acetate, 16 mg estradiol USP and 29 mg tylosin tartrate (Component TE-IS; Elanco Animal Health, Indianapolis, IN, USA). Morbidity was assessed daily throughout the course of the study and steers were treated (first treatment = florfenicol and flunixin meglumine, Resflor Gold; Merck Animal Health; and second treatment = tulathromycin, Draxxin; Zoetis) by farm personnel if cattle displayed visual symptoms (nasal discharge, cloudy eyes, drooping heads, etc.) and rectal temperature ≥40°C.

**Table 1:** Ingredient composition of common diet fed to steers from day 1 to 57

DM, %	60
Ingredient, % DM basis	
Sweet bran^1^	35
Corn silage	30
Dried distillers grains with solubles	18.04
Dry-rolled corn	15
Limestone	1.5
Salt	0.31
Rumensin^2^	0.015
Vitamins A and E premix^3^	0.11
Trace mineral premix^4^	0.024
Analyzed composition^5^	
CP	18.0
NDF	28.9
Ether extract	4.1
Calculated composition^6^	
Net energy for gain, Mcal/kg	1.29

1 Branded wet corn gluten feed (Cargill Corn Milling, Blair, NE, USA).

2 Provided monensin (Elanco Animal Health, Greenfield, IN, USA) at 27 g/ton.

3 Provided 2200 IU vitamin A and 25 IU vitamin E/kg diet.

4 Provided per kilogram of diet DM: 10 mg of Cu, 30 mg of Zn, 20 mg of Mn, 0.5 mg of I, 0.1 mg of Se and 0.1 mg of Co all from inorganic sources.

5 Based on the total mixed ration analysis from Dairyland Laboratories.

6 Based on the National Academies of Sciences, Engineering, and Medicine (2016) reported net energy for gain values of feedstuffs.

### Sample collection and analytical procedures

Weekly TMR samples were collected for DM determination and subsamples dried in a forced air oven at 70°C for 48 h. These DM values were used to calculate daily steer DMI based on individual as-fed intakes recorded by the GrowSafe system. Dried TMR samples were ground to pass through a 2-mm screen in a Retsch ZM 100 grinding mill (Retsch GmbH, Haan, Germany) and composited for analysis of nitrogen (CP; Association of Official Analytical Chemists [AOAC], 1999a; method 990.03), NDF (AOAC, 2005; method 2002.04) and ether extract (AOAC, 1999b; method 920.39) by a commercial laboratory (Dairyland Laboratories, Inc., Arcadia, WI, USA); and the analyzed compositions are presented in Table 1.

Three steers per pen (12 steers/treatment) were selected as sampling animals for blood and liver collection at all time points. Selection criteria for sampling animals included disposition and health status; steers that displayed aggression, unwillingness to enter the hydraulic chute or prior illness were excluded. Blood was collected via jugular venipuncture into vacuum tubes (serum, #366430; sodium heparin, #367874; Becton Dickinson, Franklin Lakes, NJ, USA) on day 0 (prior to pre-transit treatment injections), 1 (prior to post-transit treatment injections), 2 and 7. Serum samples were allowed to clot at room temperature for 90 min prior to centrifugation at 1500×**g** for 20 min at 4°C; serum was aliquoted into microcentrifuge tubes and stored at −80°C for future analysis of haptoglobin (**HP**) or −20°C for non-esterified fatty acids (**NEFAs**). Serum HP concentrations were analyzed using a bovine-specific ELISA kit (Hapt-11; Life Diagnostics Inc., West Chester, PA, USA; intra-assay CV = 4.8%, inter-assay CV = 12.4%) and NEFA concentrations were analyzed using a commercially available colorimetric kit (Wako Diagnostics, Mountain View, CA, USA; intra-assay CV = 5.4%, inter-assay CV = 6.0%). Sodium heparin tubes were centrifuged at 1000×**g** for 20 min at 4°C; plasma was then removed, aliquoted into microcentrifuge tubes and stored at −80°C for future analysis of ascorbate, ferric-reducing antioxidant potential (**FRAP**) and malondialdehyde (**MDA**). To prevent degradation of ascorbate, plasma for ascorbate analysis was stabilized with diethylenetriaminepentaacetic acid prior to freezing, and all samples were assayed for ascorbate within 45 days of sample collection. Commercially available kits were used for analysis of plasma total (oxidized and reduced) ascorbate (#700420; Cayman Chemical, Ann Arbor, MI, USA; intra-assay CV = 3.5%, inter-assay CV = 9.0%), FRAP (#K043-H1; Arbor Assays, Ann Arbor, MI, USA; intra-assay CV = 1.4%, inter-assay CV = 10.6%) and MDA (#700870, Cayman Chemical; intra-assay CV = 10.3%, inter-assay CV = 12.0%) concentrations.

Liver biopsies were performed on day 2 on the 36 steers selected as sampling animals using a method previously described by Engle and Spears (2000). Liver samples were snap frozen in liquid nitrogen and transported to the laboratory on dry ice, then stored at −80°C. Samples were ground in liquid nitrogen, 50 mg (wet weight) of ground liver was weighed (in duplicate) in microcentrifuge tubes for ascorbate analysis via gas chromatography-MS (**GC-MS**; Model 6890 GC coupled to Model 5975 MS; Agilent Technologies, Santa Clara, CA, USA) at the W.M. Keck Metabolomics Research Laboratory (Ames, IA, USA). Briefly, internal standards (ribitol and nonadecanoic acid) and cold extraction solvent (8 : 2 methanol–water) were added to microcentrifuge tubes prior to homogenization on a bead mill, followed by sonication for 5 min and centrifugation for 7 min at 13 000×**g**. The supernatant was put into an amber GC-MS vial and dried on a speed vacuum overnight prior to derivatization via sialylation for 30 min at 60°C. Samples were protected from light as much as possible throughout preparation and analysis. Sample separation was obtained on an Agilent-HP5MSI (30 m long, 0.250 mm ID, 0.25 μm film thickness) column. The oven program was as follows: initial temperature of 70°C for 1 min followed by 25°C/min ramp to 150°C and 15°C/min ramp to 220°C followed by 25°C/min ramp to 320°C with a final hold for 6 min. The inlet and the interface temperatures were maintained at 280°C. Detection mass range was set from 40 to 800 m/z and the GC-MS was controlled by the Agilent ChemStation software. Ascorbate was identified using the total ion mass spectrum and comparison to the National Institute of Standards and Technology (**NIST**) mass spectral library (NIST, 2014). Ascorbate was quantified by comparing the ascorbate response observed in the samples to an ascorbate standard line generated using the same method as described previously. Liver samples were prepared by block and analyzed on two different GC-MS runs within 1 week.

### Statistical analysis

Cattle growth performance and liver ascorbate concentrations were analyzed as a randomized complete block design using the Mixed Procedure of SAS 9.4 (SAS Institute Inc., Cary, NC, USA) with steer as the experimental unit and the fixed effects of treatment and block. One steer from PRE was removed from all performance analyses due to the possible electronic identification failure and subsequent invalidity of DMI data. Day 0 BW was utilized as a covariate in the analysis of mid- and final BW, and average steer DMI from day −7 through −1 was utilized as a covariate in the analysis of subsequent DMI data. Blood measures from day 0, 1, 2 and 7 were analyzed as repeated measures with the fixed effects of treatment and block and the repeated effect of day. The treatment by day interaction term was removed from the model if *P* > 0.30. The compound symmetry covariance structure was utilized for analysis of FRAP and plasma ascorbate data, while the heterogeneous autoregressive covariance structure was utilized for analysis of MDA, NEFA and HP data based on the lowest Akaike’s information criterion (Littell *et al.*, 1998). Day 0 NEFA concentrations were used as a covariate in the analysis of NEFA concentrations on subsequent sampling days. Data were tested for normality using the Shapiro–Wilk’s test; serum NEFA and HP concentrations were natural log transformed to meet the assumption of normality, and back transformed means and SEM are presented. Data were tested for outliers using Cook’s D statistic and removed if Cook’s D ≥ 0.50; based on this threshold, data from one steer were removed from analysis of FRAP on all sampling dates. Data are presented as least square means ± SEM and the differences between means were determined using the PDIFF statement in SAS. Significance was declared at *P* ≤ 0.05 and tendencies from 0.05 < *P* ≤ 0.10.

## Results

### Feedlot performance

Pre-transit (day 0) and post-transit (day 1) BW as well as percentage BW shrink after the 18-h transit event were not affected by injectable VC treatment (Table 2; *P* ≥ 0.28). Steers that received injectable VC pre-transit had the greatest BW at the midpoint of the trial (day 30/31; *P* = 0.03) and tended to have the greatest final BW (day 56/57; *P* = 0.07). Steer ADG was not affected by treatment from day 1 to 7 or 31 to 57 (*P* = 0.25). However, PRE-steers exhibited the greatest ADG from day 7 to 31 (*P* = 0.05), resulting in greater ADG by PRE-steers overall (day 1 to 57; *P* = 0.02). Steer DMI was not affected by injectable VC from day 1 to 7 (*P* = 0.13). However, there was a tendency for greater DMI by PRE- or POST-steers compared to CON-steers from day 7 to 31 (*P* = 0.08). From day 31 to 57, PRE- or POST-steers exhibited greater DMI compared to CON-steers (*P* = 0.01), resulting in greater DMI by these steers overall (*P* = 0.02). Injectable VC did not affect G:F throughout the trial (*P* ≥ 0.18).

**Table 2:** Effect of injectable vitamin C treatment on feedlot performance by beef steers

		Treatment^1^				Treatment
		CON	PRE	POST	SEM^2^	*P* value
Steers (*n*)		24	23	24		
Day 0 BW, kg		354	357	355	3.7	0.83
Day 1 BW, kg		334	335	334	3.5	0.97
Shrink^3^, %		5.7	6.2	6.0	0.23	0.28
Mid BW^4^, kg		390^b^	395^a^	389^b^	1.6	0.03
Final BW^4^, kg		429^y^	436^x^	430^y^	2.4	0.07
Average daily gain, kg/day						
1 to 7		1.43	1.78	1.34	0.198	0.25
7 to 31		1.94^b^	2.10^a^	1.94^b^	0.054	0.05
31 to 57		1.48	1.61	1.59	0.059	0.25
1 to 57		1.67^b^	1.84^a^	1.72^b^	0.042	0.02
Dry matter intake^5^, kg/day						
1 to 7		8.6	8.9	9.0	0.14	0.13
7 to 31		9.2^y^	9.6^x^	9.7^x^	0.18	0.08
31 to 57		9.3^b^	10.0^a^	9.8^a^	0.17	0.01
1 to 57		9.0^b^	9.5^a^	9.5^a^	0.14	0.02
Gain–feed, kg/kg						
1 to 7		0.164	0.198	0.150	0.022	0.31
7 to 31		0.213	0.218	0.202	0.006	0.18
31 to 57		0.161	0.160	0.164	0.006	0.89
1 to 57		0.187	0.193	0.183	0.004	0.30

Day 0 BW = BW collected pre-transit; day 1 BW = BW collected post-transit; mid BW = average BW collected on day 30 and 31; final BW = average BW collected on day 56 and 57.

1 CON = 20 ml of saline administered intramuscularly (IM) immediately prior to and post-transit; PRE = 20 ml of sodium ascorbate (250 mg/ml) administered IM immediately prior to transit and 20 ml of saline immediately post-transit; POST = 20 ml of saline administered IM immediately prior to transit and 20 ml of sodium ascorbate immediately post-transit.

2 Highest SEM of any treatment reported.

3 Percentage BW shrink after an 18-h (1675 km) transit event; a 6% shrink was applied to subsequent BW to be comparable to the post-transit BW conditions.

4 Day 0 BW utilized as a covariate in analysis.

5 Average steer DMI from day −7 through −1 utilized as a covariate in analysis.

^a,b^Values with unlike superscripts differ (*P* ≤ 0.05).

^x,y^Values with unlike superscripts tend to differ (*P* ≤ 0.10).

### Liver and blood metabolites

**Figure 1:**
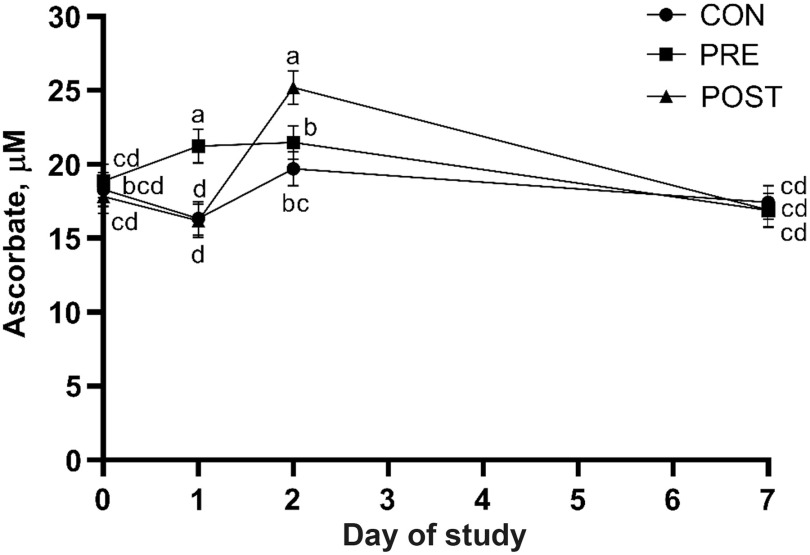
Effect of injectable vitamin C treatment and day of sampling relative to an 18-h (1675 km) transit event (treatment × day; *P* < 0.01) on plasma ascorbate concentrations of beef steers based on repeated measures analysis of samples collected on day 0 (pre-transit), 1 (post-transit), 2 and 7; values with unlike superscripts differ (*P* ≤ 0.05) across treatments and sampling days. CON = 20 ml of saline administered intramuscularly (IM) immediately prior to and post-transit; PRE = 20 ml of sodium ascorbate (250 mg/ml) administered IM immediately prior to transit and 20 ml of saline immediately post-transit; POST = 20 ml of saline administered IM immediately prior to transit and 20 ml of sodium ascorbate immediately post-transit.

**Figure 2:**
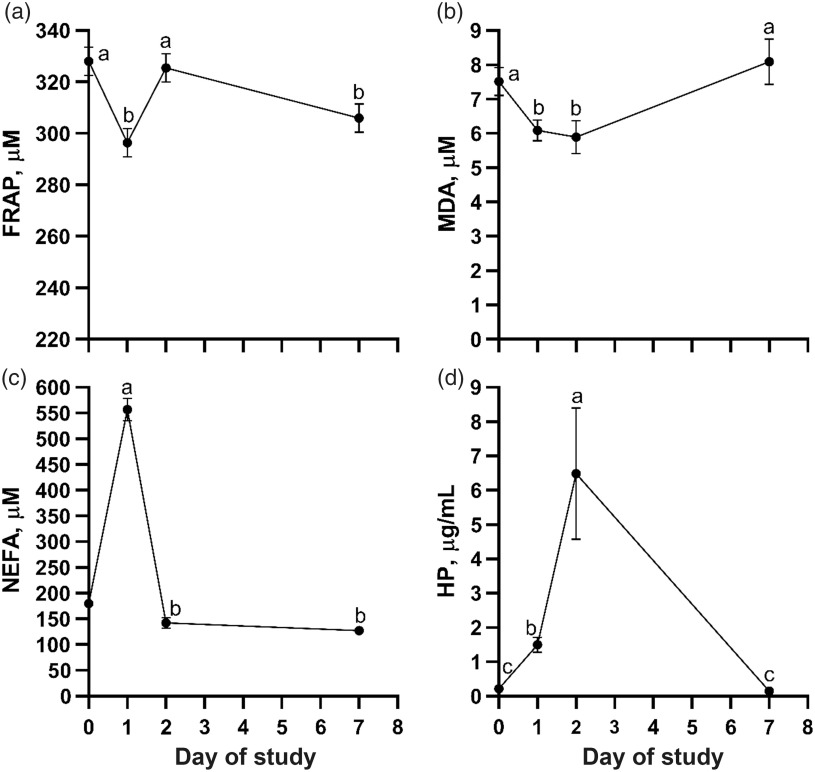
Effect of day (*P* < 0.01) of sampling relative to an 18-h (1675 km) transit event on plasma ferric-reducing antioxidant potential (FRAP; panel a), plasma malondialdehyde (MDA; panel b), serum non-esterified fatty acid (NEFA; panel c) and serum haptoglobin (HP; panel d) concentrations of beef steers based on repeated measures analysis of samples collected on day 0 (pre-transit), 1 (post-transit), 2 and 7. Values within a panel with unlike superscripts differ (*P* ≤ 0.05) across sampling days; day 0 values were used as a covariate in analysis of serum NEFA concentrations.

Treatment means for liver and blood metabolites are presented in Table 3. Liver ascorbate concentrations on day 2 were not affected by treatment (*P* = 0.11). Based on repeated measures analysis of blood samples collected on day 0, 1, 2 and 7, there were no overall treatment effects for the blood metabolites measured (*P* ≥ 0.21). However, a treatment × day effect was observed for plasma ascorbate (*P* < 0.01) and day effects were observed for the remaining metabolites (*P* < 0.01). Pre-transit (day 0) plasma ascorbate concentrations were similar among treatments, but steers that received injectable VC pre-transit had the greatest plasma ascorbate concentrations post-transit (day 1), while steers that received injectable VC post-transit had the greatest concentrations on day 2 (Figure 1). Plasma ascorbate concentrations were similar to pre-transit values on day 7 for all treatments. Plasma FRAP concentrations were decreased post-transit (day 1) before returning to pre-transit values on day 2 and decreasing again on day 7 (Figure 2a). Plasma MDA concentrations were decreased on day 1 and 2 before returning to pre-transit values on day 7 (Figure 2b). Alternatively, serum NEFA concentrations increased sharply on day 1 before returning to pre-transit values by day 2 (Figure 2c). Serum HP concentrations were greatest on day 2, intermediate on day 1 and similar to pre-transit values by day 7 (Figure 2d).

**Table 3:** Effect of injectable vitamin C treatment on day 2 liver ascorbate concentrations and blood parameters of beef steers

	Treatment^1^				Treatment
	CON	PRE	POST	SEM^2^	*P* value
Liver ascorbate, µg/g wet tissue	183	225	253	23.1	0.11
Blood parameters^3^					
Plasma FRAP, µM	309	313	319	7.3	0.59
Plasma MDA, µM	6.9	6.8	6.9	0.47	0.97
Serum NEFA^4^^,^^5^, µM	212	215	221	12.4	0.87
Serum HP^5^, µg/ml	0.94	0.80	0.53	0.225	0.21

FRAP = ferric-reducing antioxidant potential; HP = haptoglobin; MDA = malondialdehyde; NEFA = non-esterified fatty acid.

1 CON = 20 ml of saline administered intramuscularly (IM) immediately prior to and post-transit; PRE = 20 ml of sodium ascorbate (250 mg/ml) administered IM immediately prior to transit and 20 ml of saline immediately post-transit; POST = 20 ml of saline administered IM immediately prior to transit and 20 ml of sodium ascorbate immediately post-transit.

2 Highest SEM of any treatment reported.

3 Based on repeated measures analysis of blood samples collected on day 0, 1, 2 and 7; day effects are shown in Figure 2.

4 Day 0 concentrations utilized as a covariate in analysis.

5 Data were natural log transformed prior to analysis.

## Discussion

Transit involves psychological stress, food and water deprivation as well as muscle fatigue from standing for long periods of time. These physiological responses can stimulate inflammation (Marques *et al.*, 2012) and increase the production of reactive oxygen species (Piccione *et al.*, 2012) which may lead to oxidative stress if cellular antioxidants are overwhelmed. Inflammation and oxidative stress are nutritionally demanding processes due to the need for amino acids to synthesize acute phase proteins and antioxidants as well as ATP to repair or degrade oxidatively damaged molecules (Grune *et al.*, 1997; Griffith, 1999). Therefore, this study sought to investigate proactive (pre-transit) *v*. reactive (post-transit) administration of a powerful antioxidant, VC. It was hypothesized that a pre-transit VC injection would dampen transit-induced inflammation and oxidative stress and thus spare energy and protein for growth.

Although some species require exogenous VC, cattle possess a functional form of l-gulonolactone oxidase which allows for the endogenous synthesis of VC from glucose in the liver (Bánhegyi *et al.*, 1997). However, management practices that impose stress on cattle may increase the requirement and exceed biosynthetic capacity of VC to combat oxidative stress. Prior to transit, plasma ascorbate concentrations of steers in the current study were 18.3 ± 3.7 µm/l, which falls within the reference range (17.1 to 28.2 µm/l) for beef cattle proposed by Matsui (2012). Transit decreased plasma ascorbate concentrations by 10% in steers that did not receive a VC injection pre-transit (CON and POST; 16.3 ± 3.3 µm/l). Other production stressors have also been shown to decrease VC status in cattle. Padilla *et al.* (2006) observed a 51% decrease in plasma ascorbate concentrations when lactating Holstein cows were exposed to heat stress conditions (24°C on the first day, 26°C on the second day and 28°C for the next 12 days; 8.7 µm ascorbate/l) *v*. control conditions (18°C for 14 days; 17.7 µm ascorbate/l). Cummins and Brunner (1991) reported 17% lesser plasma ascorbate concentrations in male Holstein calves housed in metal pens *v*. calves housed in commercial calf hutches (24.4 *v*. 29.5 µm/l); metal pens are considered to be a more stressful form of housing based on increases in plasma cortisol concentrations. Cortisol concentrations were not measured in the current study, so it is unclear whether there were treatment differences in this stress hormone; however, post-transit increases in cortisol concentrations of cattle have been well-documented (Crookshank *et al.*, 1979; Kent and Ewbank, 1983; Marques *et al.*, 2012). Stress associated decreases in plasma VC may be a result of increased uptake by the adrenal glands to support catecholamine biosynthesis (Kipp and Rivers, 1987) or by other tissues where there is an increased metabolic demand for VC. In contrast to CON- and POST-steers, PRE-steers had 13% greater plasma ascorbate concentrations immediately post-transit relative to pre-transit concentrations. On day 2, approximately 24 h after post-transit treatment injections, POST-steers exhibited a 56% increase in plasma ascorbate concentrations relative to concentrations measured immediately after unloading. Additionally, plasma ascorbate concentrations of CON-steers increased by 21% on day 2 relative to post-transit concentrations, suggesting endogenous synthesis of VC may have been upregulated as these steers were not given exogenous VC. Liver and plasma ascorbate concentrations on day 2 followed a similar trend with the greatest concentrations observed for POST-steers and the least concentrations observed for CON-steers, with PRE-steers being intermediate.

Although plasma ascorbate concentrations returned to pre-transit values and were not different among treatments by day 7, effects of injectable VC on feedlot performance were observed later in the post-transit period. Steers that received VC pre-transit were 6 kg heavier than POST-steers and 7 kg heavier than CON-steers at the end of the 56-day post-transit period. Not surprisingly and regardless of treatment, steer ADG was greatest during the period immediately following the administration of an anabolic implant (day 7 to 31; 1.99 kg/day) *v*. later in the trial (day 31 to 57; 1.56 kg/day). However, the ADG advantage for steers that received a pre-transit VC injection was only observed from day 7 to 31. In addition to antioxidant protection, VC is intimately involved in collagen biosynthesis and remodeling (Archile-Contreras and Purslow, 2011) which are processes that support skeletal muscle hypertrophy by facilitating migration of satellite cells (Nishimura *et al.* 2008). It is also interesting to note that despite steers who received VC at any time point having greater DMI than CON-steers, only steers that received VC pre-transit exhibited a growth response. This could indicate that PRE-steers were able to utilize dietary nutrients for growth, while POST-steers may have utilized those nutrients to replenish protein and energy stores depleted during the transportation event. Another potential mechanism to explain the growth response to injectable VC is the role of VC as a cofactor in enzymes involved in carnitine biosynthesis. Carnitine is essential for transporting fatty acids into the mitochondria for catabolism and subsequent production of ATP. In the current study, post-transit serum NEFA concentrations were 210% greater than pre-transit values likely due to the increased lipolysis stimulated by food deprivation (glucagon) and psychological stress (cortisol). Therefore, it is possible that there was an increased need for carnitine to utilize these released fatty acids for energy production and growth. Further research is needed to determine whether VC-dependent enzymes involved in collagen and carnitine biosyntheses could be influencing the growth response observed herein.

Despite treatment differences in plasma concentrations of VC, a known cellular antioxidant, there was no effect of treatment on total plasma antioxidant capacity measured by FRAP. Although FRAP was likely not capturing changes in plasma VC due to degradation in these samples, injectable VC treatments could have affected other antioxidants measured by FRAP, such as α-tocopherol. Circulating antioxidant capacity has been shown to decrease in response to transit stress. Chirase *et al.* (2004) observed a 9.5% decrease in total antioxidant capacity of crossbred beef steers (207 ± 21 kg) after a 19 h 40 min (1930 km) transit event. Similarly, antioxidant capacity in the current study was decreased 9.7% immediately post-transit relative to pre-transit values. While Chirase *et al.* (2004) reported a sustained decrease in antioxidant capacity through day 28 post-transit, antioxidant capacity returned to pre-transit values by day 2 in the current study. These conflicting results could be because the steers utilized by Chirase *et al.* (2004) were multi-sourced and assembled at an order buyer barn before transportation to the Texas A&M University beef research facility, while steers utilized in the current study had been previously adapted to the diet and environmental conditions at the Iowa State University Beef Nutrition Farm before the transportation event. Chirase *et al.* (2004) also reported a threefold increase in serum MDA, a marker of lipid peroxidation, after transit, while the current study observed lesser plasma MDA concentrations on day 1 and 2 post-transit. This discrepancy may be explained by differences in analytical methods used to determine MDA as Chirase *et al.* (2004) utilized HPLC which is more specific for MDA than the colorimetric method utilized in the current study (Del Rio *et al.*, 2005).

Transit induced an inflammatory response in the current study evidenced by increased concentrations of the acute phase protein HP. It was hypothesized that a pre-transit VC injection would mitigate the decline in antioxidant status and subsequently decrease inflammation; however, no treatment effects were observed for serum HP concentrations. Urban-Chmiel *et al.* (2011) also observed no effect of injectable VC on HP concentrations 3, 7, 14 and 21 days after Simmentaler calves (100 to 120 kg) received a subcutaneous VC injection (1.25 g/steer; 10.4 to 12.5 mg/kg BW) on the first and second days in the feedlot. Increased HP concentrations after transportation and arrival at the feedlot have been positively correlated with bovine respiratory disease (**BRD**; Godson *et al.*, 1996; Joshi *et al.*, 2018). Several studies have investigated the use of VC as an ancillary therapy at the time of BRD treatment due to the antioxidant role of VC in protecting phagocytic immune cells from the reactive oxygen species these cells produce to kill infectious pathogens. Although the effectiveness of VC as an ancillary BRD therapy has not been definitively proven, according to a survey conducted by the National Animal Health Monitoring System, approximately one of three cattle affected and treated for BRD in large feedlots (≥1000, animals) is given a VC injection as part of an initial course of treatment for respiratory disease (United States Department of Agriculture, 2013). No effects of injectable VC on respiratory disease rates were observed in the current study (data not shown), likely due to the most respiratory treatments occurring within 8 days of arrival (52 days prior to trial initiation and VC injection).

Vitamin C is a relatively ignored nutrient in beef production due to the lack of a perceived dietary requirement. In the current study, a pre-transit VC injection mitigated the transit-induced decline in plasma ascorbate concentrations and improved post-transit feedlot performance of beef steers. Due to the differential responses observed for pre- *v*. post-transit administration, the timing of VC administration is likely an important consideration for beef producers looking to adopt this supplementation strategy. Future research should seek to determine how dose and timing of injectable VC administration affect cattle health and performance.

## Software and data repository resources

These data were not deposited in an official repository.
